# Biosciences Proposal Bootcamp: Structured peer and faculty feedback improves trainees’ proposals and grantsmanship self-efficacy

**DOI:** 10.1371/journal.pone.0243973

**Published:** 2020-12-28

**Authors:** Crystal M. Botham, Shay Brawn, Latishya Steele, Cisco B. Barrón, Sofie R. Kleppner, Daniel Herschlag

**Affiliations:** 1 Stanford Biosciences Grant Writing Academy, Stanford University, Stanford, California, United States of America; 2 Program in Writing and Rhetoric, Stanford University, Stanford, California, United States of America; 3 Office of Graduate Education, Stanford University, Stanford, California, United States of America; 4 Office of Postdoctoral Affairs, Stanford University, Stanford, California, United States of America; 5 Department of Biochemistry, ChEM-H Institute, Department of Chemistry, Stanford University, Stanford, California, United States of America; University of Texas MD Anderson Cancer Center, UNITED STATES

## Abstract

Grant writing is an essential skill to develop for academic and other career success but providing individual feedback to large numbers of trainees is challenging. In 2014, we launched the Stanford Biosciences Grant Writing Academy to support graduate students and postdocs in writing research proposals. Its core program is a multi-week Proposal Bootcamp designed to increase the feedback writers receive as they develop and refine their proposals. The Proposal Bootcamp consisted of two-hour weekly meetings that included mini lectures and peer review. Bootcamp participants also attended faculty review workshops to obtain faculty feedback. Postdoctoral trainees were trained and hired as course teaching assistants and facilitated weekly meetings and review workshops. Over the last six years, the annual Bootcamp has provided 525 doctoral students and postdocs with multi-level feedback (peer and faculty). Proposals from Bootcamp participants were almost twice as likely to be funded than proposals from non-Bootcamp trainees. Overall, this structured program provided opportunities for feedback from multiple peer and faculty reviewers, increased the participants’ confidence in developing and submitting research proposals, while accommodating a large number of participants.

## Introduction

Apart from the obvious financial benefits of submitting a successful grant proposal, the proposal writing process provides graduate students and postdocs with skill-building opportunities for thinking critically and communicating ideas, required competencies for most careers [1]. Writing a high-quality proposal requires the proposal writer to develop an in-depth understanding of the primary literature; to identify important problems or critical barriers to progress in their field; to evaluate strategy, methodology, and analyses to accomplish the specific aims of the project; and to articulate how the proposed research challenges or seeks to shift current research paradigms [2, 3]. However, precisely because it places these intellectual demands on the writer, writing a proposal is a time-consuming process: it requires the writer to devote time to reading the primary literature and thinking deeply about their research questions, approaches to answer those questions, and implications of their research on the broader field. Additionally, a well-reasoned proposal requires considerable time to develop as well as refine ideas and their presentation, which is aided by opportunities to obtain and incorporate feedback.

Unfortunately, the potential intellectual and professional development embodied in the grant writing process are often under-realized. Opportunities for graduate students and postdocs to learn how to write proposals are highly variable. Many rely on their primary mentor/supervisor to provide informal training and feedback on their proposals. However, feedback from mentors can vary widely, with some mentors providing too little feedback and others significantly rewriting drafts, which limits the iterative process (draft-feedback-revision). Formal, large-scale grant writing seminars typically cover the agency’s guidelines, application logistics, and tips on writing techniques but yield uneven results and low improvement in writing capability. These programs can underemphasize the power of an iterative process in writing a compelling proposal. Conversely, programs that can provide the critical and iterative feedback that is required for persuasive writing often have small numbers (<30) of participants and are discipline specific [4–6].

To create opportunities for large-scale formal training and individualized feedback for graduate students and postdocs writing research proposals, we launched the Stanford Biosciences Grant Writing Academy in 2014. At the core of the Grant Writing Academy is an eight-week intensive Proposal Bootcamp course designed to provide substantial and substantive feedback as writers develop and refine their proposals. The Proposal Bootcamp—combining structured peer review using rubrics, faculty feedback, written guidelines for grant writing, and short informational lectures on aspects of grant writing—was designed to maximize the benefits of formative assessment and peer review.

Formative assessment, feedback given while work is in progress as to reinforce learning and aid development, is important to improving learning outcomes [7–10]. The literature on formative assessment also suggests that it can play a crucial role in fostering self-regulated learning, which is important for the development of lifelong learners [11, 12]. One important mediator for the impact of formative assessment is the use of rubrics, which have been determined in multiple studies to have a positive effect on learning outcomes [13]. Rubrics are thought to improve students’ understanding, increase their confidence, and help them internalize the goals and values of the task in which they are engaged [13].

Unlike peer review as it is understood in the vetting of manuscripts for publication or grants, peer review in the context of writing pedagogy involves feedback and assessment shared reciprocally among co-learners rather than delivered from experts. One concern that students often have about peer review is that they and their peers may not be expert enough in the subject domain to provide useful feedback to each other [14]. However, studies show that experience with peer review raises students’ confidence in both their own and their peers’ assessments [14]. Indeed, as demonstrated by a series of studies by Cho et al., subject expertise is not a prerequisite for effective feedback and feedback from peers can actually be more helpful for students than that from subject matter experts. Students who received feedback from a single expert revised less effectively than students who received feedback from multiple peers [15]. This difference may partly arise because students receiving feedback from multiple peers received significantly more feedback than those receiving feedback from single experts. The feedback was also qualitatively different: experts tended to be more directive in their feedback, while peers were more non-directive [16]. In a later study, Cho et al. found that students who received feedback from a single expert tended to make more simple repairs (mechanics, grammar, factual error) in revision, which are not associated with overall improvement in quality; by contrast, students who received feedback from multiple peers made more complex revisions (elaboration, clarification, support), which is associated with overall improvement in quality [17]. Indeed, even the prospect of peer assessment seems to dramatically improve performance on a learning task [18].

While subject area training is not necessary to successfully peer review, training in the practice of effective peer review itself can be an important mediator in the success of peer review [14, 19–21]. Additionally, peer review benefits the reviewer as it allows them to see a broader variety of exemplars and to more deeply internalize the evaluation criteria [13]. It has the added benefit of asking the peer reviewer to ‘think like a reviewer,’ which can strengthen their own writing [22]. Poe et al. [23] described peer review as contributing to the student’s understanding of scientific writing as a persuasive endeavor by putting students in the role of reviewers. One study found that students who only gave peer feedback revised more effectively than students who only received peer feedback [24].

Since 2014, the annual Proposal Bootcamp has provided 525 doctoral students and postdoctoral fellows/scholars with multi-level feedback (peer and faculty). Importantly, the applicant success rate for our Proposal Bootcamp participants was 39%, nearly double the rate for non-Bootcamp trainees. Participants reported improved quality of proposals and increased confidence in grant writing abilities. The faculty review workshops increased faculty engagement in the proposal development process and were valued by both the Bootcamp participants (97% “Agreed” workshops were important) and faculty (98% rated workshops as “Excellent” or “Good”). The Proposal Bootcamp can be implemented at other universities to support National Institutes of Health (NIH) (e.g., NIH fellowships and career development awards, etc.) and other (e.g., National Science Foundation, NSF, etc.) proposals.

## Materials and methods

The Stanford School of Medicine Senior Associate Dean of Graduate Education and Postdoctoral Affairs (Daniel Herschlag, PhD), Assistant Dean of the Office of Postdoctoral Affairs (Sofie Kleppner, PhD), Assistant Dean of Graduate Education and Diversity (Terrance Mayes, PhD), and Director of Strategic Research Development in the Division of Cardiovascular Medicine (Crystal Botham, PhD) created the Biosciences Grant Writing Academy in 2014. The Grant Writing Academy goals were to support graduate students and postdocs in writing research proposals.

The annual multi-week Proposal Bootcamp (now 8 weeks) in the Autumn Quarter is one of the core programs overseen by the part-time (40%) Grant Writing Academy Director (Crystal Botham, PhD; a research development strategist). The Grant Writing Academy also has a part-time administrator who helps with operations including the faculty review workshop signups, reminder emails, advertising, and printing handouts. The Proposal Bootcamp’s weekly meetings with peer review and focused faculty feedback at faculty review workshops were based on a course called *Tackling Your K* taught by Crystal Botham through the Cardiovascular Institute at Stanford. The *Tackling Your K* course typically included 6–8 postdocs, but training postdocs as grant coaches has enabled us to scale up the Bootcamp to serve roughly 100 participants per course. The Senior Associate Dean for Graduate Education and Postdoctoral Affairs in the School of Medicine provides the Grant Writing Academy’s budget.

### Proposal Bootcamp logistics

Two to three months before each Proposal Bootcamp, we emailed potential participants using listservs organized by the Stanford Office of Postdoctoral Affairs and the Biosciences Office of Graduate Education. Graduate Students in the Biosciences Program were encouraged to enroll in the Bootcamp in their second or third year of training. We also posted fliers advertising the upcoming Proposal Bootcamp in buildings housing the Stanford School of Medicine and Biosciences programs. We stressed to prospective participants that they must be actively working on a proposal. Almost all graduate students enrolled in the Proposal Bootcamp as a two-unit Writing Compelling Fellowships and Career Development Awards (BIOS 242) course, which was approved by the School of Medicine Registrar Office as Satisfactory/No Credit grading.

Bootcamp participants attended two-hour weekly meeting between August 25 and November 21, 2014 (2014 Cohort); September 21 and November 20, 2015 (2015 Cohort); September 26 and November 18, 2016 (2016 Cohort); September 25 and November 17, 2017 (2017 Cohort); September 24 and November 16, 2018 (2018 Cohort); or September 23 and November 15, 2019 (2019 Cohort). Bootcamp participants were divided into small groups of 12–25 graduate students and/or postdocs working on either fellowships, e.g., NIH F31 or F32, or career development awards, e.g., NIH K Awards. The weekly meetings were led by grant coaches, postdocs we hired and trained as course teaching assistants, and consisted of a short mini lecture and peer review. Bootcamp participants also joined up to two faculty review workshops to receive feedback from faculty.

### Mini lecture topics

During the first meeting, grant coaches provided initial training in giving effective feedback and reviewed the course’s effective feedback guidelines with Bootcamp participants. Participants were reminded of these guidelines during each subsequent meeting. The guidelines drew from a handout from Stanford’s Hume Center for Writing and Speaking [25] and reflected widely recognized best practices in giving feedback on drafts [26]. In this model, feedback should be:

- **Prioritized** (more important issues first). When writers receive too much feedback, with as much emphasis placed on minor grammatical issues as on major conceptual flaws, they can be overwhelmed and are more likely to work on the simplest problems first rather than tackling major issues.
- **Supportive** (respectful, attentive to the writer’s purpose). Because writers are often inclined to take feedback personally, it is important to frame feedback in ways that support the writer’s development not only by recognizing strengths but also by identifying promising areas for improvement.
- **Specific** (pointing to concrete features of the text, offering explicit reasoning). Vague feedback (such as “your writing is confusing” or “this is incoherent”) can be frustrating for writers because they may not be able to see what the issue is. Feedback that points to where and how the writing is not working is more helpful (“I was confused when you shifted from talking about A to talking about B” or “The connection between these two sentences is not clear.”)
- **Descriptive or questioning** (reader-based, non-judgmental). The way feedback is framed can have a big impact on how it is received by the writer. In the context of peer review, it’s important not to come across as overly directive or judgmental. Thus, it is best to frame commentary in terms of the reader’s experience or to seek explanations (e.g., “When I read this, I thought at first you were saying X, but then it seemed like you were saying Y” or “Did you mean to say X or did you mean to say Y?”) instead of as advice or judgment (“Focus on X instead of Y”).
- **Future-oriented** (focused on next steps, what is possible). Focusing on the future and the potential for revision helps to create a constructive environment for mutual learning. It also reinforces an understanding of writing as a process.

During the first weekly meeting, grant coaches also discussed that the drafts and feedback were confidential and not shareable outside the classroom. Each Bootcamp participant signed a “Confidentially and Nondisclosure Rules” agreement based on the NIH peer review policy [27].

During subsequent weekly meetings, the grant coaches’ mini lectures (<15 minutes, Table 1) offered strategic advice for handling different aspects of writing grants that are applicable to a broad range of funding sources (e.g., NIH or private foundations like the American Heart Association). Topics included “Building the Research Strategy,” “Thinking Like a Reviewer,” and “Drafting Figures for Grants.”

**Table 1:** Structure of the eight-week Proposal Bootcamp. Grant coaches delivered short (<15 minute) mini lectures. During the remainder of the two-hour weekly meeting, Bootcamp participants used a structured format to peer review drafts. Before week 1, Bootcamp participants attended an in-person seminar or watched an online video about how to write the one-page Specific Aims document. Faculty review workshops took place during weeks 3–4 and weeks 5–6.

Week	Mini Lecture Topic	Document(s) Peer Reviewed
1	Course overview; Peer-review process	One-page Specific Aims
2	Building the research strategy (Significance, Innovation, Approach)	NIH-style Biosketch
3	Thinking like a reviewer	One-page Specific Aims
		Significance (and Innovation)
4	Developing career training plans	One-page Specific Aims
		Approach (1 aim only)
5	Obtaining clarity in scientific writing	Candidate’s Background
		Career Goals
6	Establishing and maintaining a writing practice	Training Plan
7	Drafting figures for grants	One-page Specific Aims
		Approach (all aims)
8	Questions and answers (no lecture)	Project Summary

### Structured peer-review format

**Fig 1:**
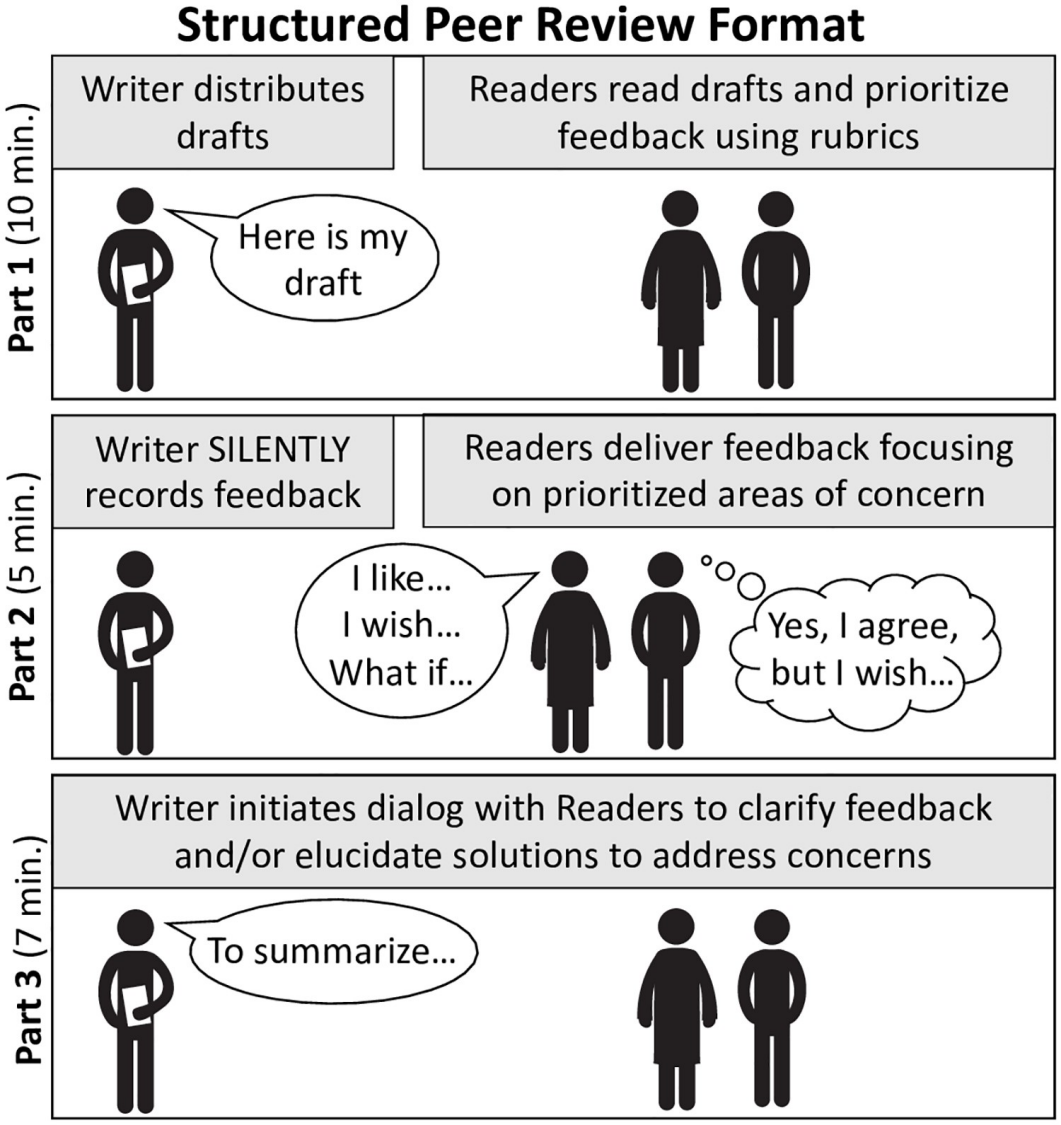
During the weekly meetings, documents were peer reviewed using a structured format. Peers participated in each peer-review role (*Writer* or *Reader*) each week, thus receiving prioritized feedback from peer reviewers. This structured format ensured fast, organized, and effective feedback.

The weekly peer-review groups were organized around the type of proposal, either fellowship-like (e.g., NIH F31/F32) or career development award-like (e.g., NIH K99/R00 or K08). Our fellowship-like groups included both graduate students and postdocs. The career development award-like groups included only postdocs. Participants within the weekly meetings had diverse training and research experiences. We did not group Bootcamp participants based on research or expertise. The participants’ documents were peer reviewed in small groups of three peers using a structured format that consisted of three parts (Fig 1):

Part 1: The *Writer* provided the document to be peer reviewed. *Reader 1* and *Reader 2* read the document and prioritized feedback using the relevant rubric (10–15 minutes).
Part 2: The *Writer* listened to the *Readers* prioritized feedback. The *Writer* was instructed to write down the feedback. *Reader 1* orally summarized their experiences of reading the draft, focusing on the three prioritized areas of concern for revision. *Reader 2* could support what *Reader 1* said and/or added suggestions for revision priorities. Ideally, *Reader 2* looked for ways to relate their feedback to what *Reader 1* said so that the *Writer* developed a coherent sense of what was needed (5–7 minutes).
Part 3: The *Writer* initiated a dialogue with the *Readers*, e.g., asking for clarification; orally summarizing feedback to confirm understanding; initiating a discussion about apparent contradictions in *Readers’* responses; and/or suggesting or soliciting potential solutions to address concerns (7–10 minutes).

To ensure effective peer review, we asked *Reader 2* to monitor time for the peer review and, if necessary, remind peers to follow the structured feedback guidelines. Three cycles of these peer reviews occurred weekly (each with Parts 1–3, Fig 1) until all peers participated in each peer-review role (e.g., *Writer*, *Reader 1*, and *Reader 2*) and received prioritized feedback from up to two peer reviewers.

This structured peer-review format was designed to maximize the benefits of peer review by creating a process for the *Reader* to prioritize their feedback and provide focused, specific feedback. We found that asking the *Writer* to remain silent during the first part of the review helped the *Writer* to let go of defensive first responses and carefully listen to what was being said. Asking the second *Reader* to synthesize their feedback with the feedback of the first *Reader minimized* the potential to overwhelm the *Writer*. This also helped to illuminate potential disagreements between *Readers* in a way that did not overburden the *Writer* with simply getting conflicting advice, and instead encouraged dialogue to help the *Writer* understand the sources of disagreement. Finally, asking the *Writer* to run the conversation after the initial round of feedback enabled the *Writer* to be in charge of the process and fostered a more forward-thinking discussion about choices the *Writer* might make in the revision.

During peer review, grant coaches monitored the small groups to ensure the structured format and effective feedback guidelines were followed. The small group members either remained consistent during the Bootcamp or changed weekly depending on attendance for the group and the grant coach’s preferences.

### Document-specific rubrics

**Fig 2:**
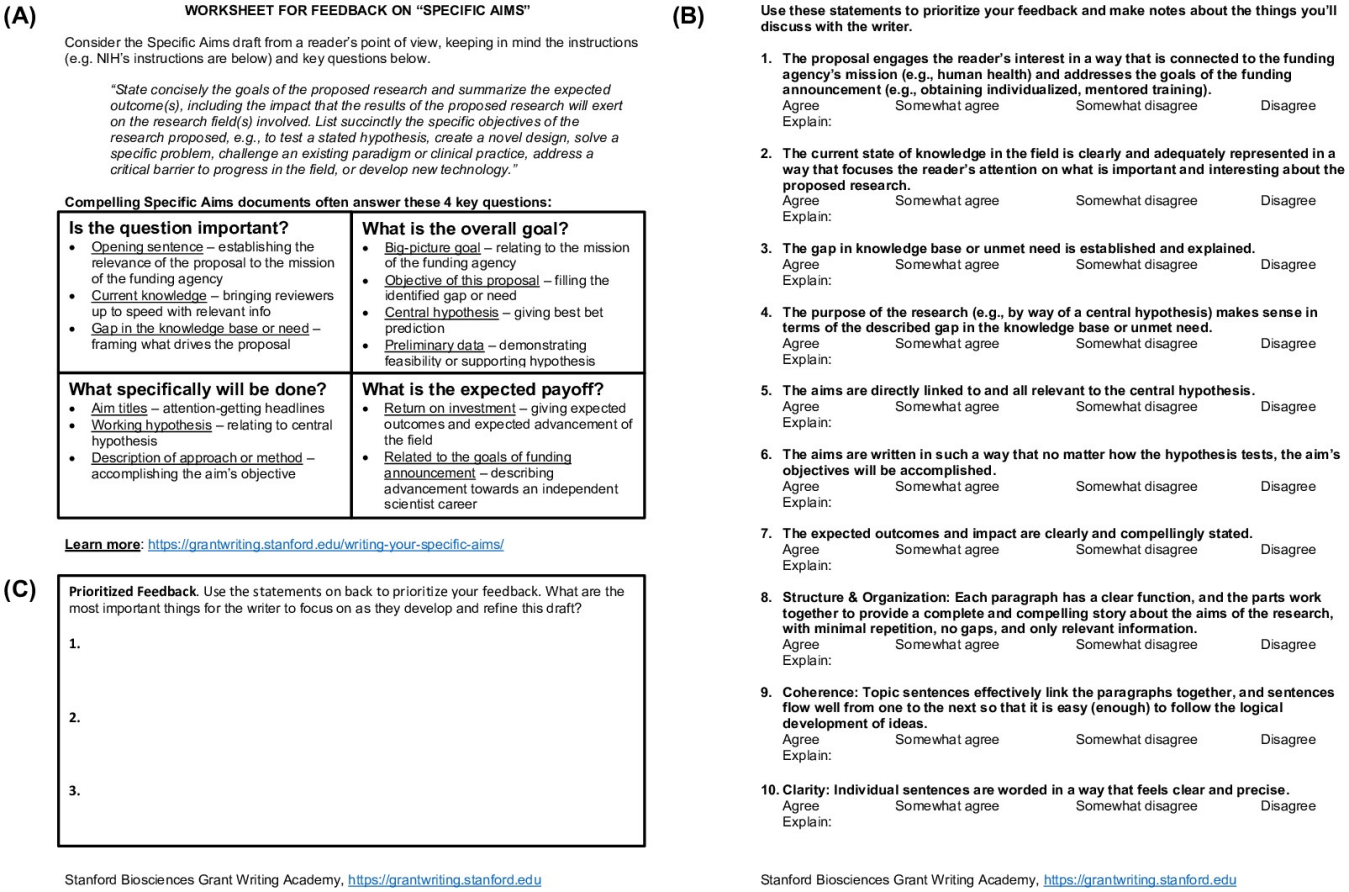
Specific aims rubric worksheet was designed to enable peers to provide prioritized feedback to the *Writer*. (A) Each worksheet provided strategic advice. (B) First, *Readers* used the back of the worksheet to rate statements that described specific aspects of the document. (C) Then, *Readers* prioritized their feedback.

We created document-specific rubric worksheets for peer reviewing the one-page Specific Aims (Fig 2), Research Strategy (S1 Fig), Career Development (S2 Fig), and NIH Biosketch (S3 Fig). We used language similar to the review criteria for the NIH National Service Research Award (NRSA) F Fellowships and K Awards, and applicable to many bioscience-focused fellowships and career development awards. The worksheets were designed to enable peers to provide prioritized feedback to the *Writer*. *Readers* used the backs of the worksheets to rate (Agree, Somewhat Agree, Somewhat Disagree, Disagree) statements that described specific aspects of a critical proposal document, e.g., one-page Specific Aims. Then, *Readers* used these statements to prioritize their feedback and provide the most important things for the *Writer* to focus on as s/he developed and refined the draft. The worksheets also provided strategic advice to consider when peer reviewing the document.

### Feedback via faculty review workshops

The Faculty Feedback Workshop provided an opportunity for Bootcamp participants to solicit focused feedback from faculty about their one-page NIH-style Specific Aims document. Each workshop lasted two hours and was attended by up to two faculty reviewers, four Bootcamp participants, and one grant coach. Bootcamp participants signed up for the workshops based on the faculty expertise or timing of the workshop, so the faculty reviewers were not necessarily subject experts on the Bootcamp participants’ research topics. At the workshops, the grant coaches provided a succinct overview of the Proposal Bootcamp, effective feedback guidelines, and outlined the structure for the faculty review workshop, which followed a similar format as the peer-review sessions: faculty reviewers provided prioritized feedback to the silent Bootcamp participant; afterward, the Bootcamp participant could initiate a dialogue to clarify feedback. No pre-workshop preparation or follow-up was required of the faculty.

We used SignUpGenius.com (a free organizing sign-up tool) to manage the faculty review workshops. Faculty signed up for a convenient workshop time. In early years, we attempted to group faculty at the workshops based on research topics, but this was logistically difficult. Additionally, we found the workshops were still extremely productive, sometimes more so, if the faculty had diverse research experiences and expertise. During the early years of the Bootcamp, we widely emailed faculty members at Stanford to encourage participation in the faculty review workshops. Later, we emailed faculty, starting six months before the start of the Bootcamp, who had already participated or who had a student or postdoc who participated in the Bootcamp. We had approximately 50 workshops available for each Bootcamp.

After the workshop, faculty received a Qualtrics survey link for sharing feedback and requesting an optional letter from the Senior Associate Dean for Postdoctoral Affairs and Graduate Education describing their service to the University (for their Faculty Actions/Promotion File). All faculty received a thank you card and a small gift ($25 gift card to the Stanford Bookstore).

### Grant coach recruitment and training

We recruited postdocs in the biosciences to be teaching assistants, called grant coaches. The grant coach application asked for a list of past proposals submitted and funded as well as a <300-word essay of interest (*Why are you interested in being a grant coach*? *What experiences and qualities will you bring to such a position*?). We also required prospective grant coaches to read a mock K Award training plan and provide written feedback that would improve the next draft. In responses, we looked for applicants that followed our best practices for giving feedback described above. We also required the immediate supervisor of the prospective grant coach to provide permission for them to participate and confirm the applicant is a strong writer and has the aptitude to help others improve their writing. Prior Proposal Bootcamp participants were encouraged to apply.

Both new and returning grant coaches were trained the summer before the annual autumn Bootcamp; training lasted six hours over three weeks in the first year and is now eight hours over five weeks. Initially, training revolved around rhetoric and the teaching of writing, providing effective written feedback, and facilitating peer review. From the start, the training involved providing coaches with firsthand experiences giving and receiving feedback using the Bootcamp guidelines. The training model has shifted increasingly toward a professional development model, offering coaches an opportunity to reflect on, revise, and augment both pedagogical and curricular aspects of the Bootcamp. In past years, the coaches developed and refined the mini lectures during training. The mini lectures (5–15 minutes) were recorded as part of a small grant from Stanford’s Office of the Vice Provost for Teaching and Learning. These videos are now integrated into the Bootcamp curriculum and made available on the Grant Writing Academy’s website for Stanford affiliates. For the last few years, coaches have read How Learning Works: Seven Research-Based Principles for Smart Teaching [28] and applied best practices to facilitate a deeper learning by Bootcamp participants. During training, coaches (1) identified specific curricular gaps in the Proposal Bootcamp and (2) developed (individually or in small groups) a specific learning modality that addressed a specific curricular gap, e.g., in coverage, lack of emphasis, underserved constituencies, or problems with outcome. Coaches peer reviewed these “augmentations” using the Bootcamp feedback guidelines. During the Bootcamp, the coaches also met weekly for one hour with the Director of the Grant Writing Academy to discuss curricular activities, classroom management, and other topics. Coaches were paid $30–42 per hour for their coaching time (typically 50–55 hours over 8 weeks), which included: two hours for the weekly meeting, one office hour, one hour for preparation each week as well as two hours for each faculty review workshop that they facilitated.

Specific roles of the grant coaches included:

- Training Bootcamp participants to give and receive feedback via a structured process at weekly meetings;
- Supporting and facilitating focused feedback from faculty at faculty review workshops;
- Clarifying and reinforcing writing strategies presented through course materials (readings, videos, lectures, class activities);
- Fostering a supportive writing community within and beyond the classroom;
- Providing constructive feedback to writers;
- Delivering the curriculum and other relevant content and sharing expertise.

### Analysis of success rates and grantsmanship self-efficacy

The number of proposals submitted and awarded, the award period (start and end dates), and the total amount requested were determined from queries to the Stanford Electronic Research Administration System in February 2020. This system records externally funded proposals, like career development awards and fellowships to the NIH (e.g., NIH K Awards and F32) or private foundations. Proposal Bootcamp participants from the 2014–2018 cohorts were compared to non-Bootcamp trainees. Non-Bootcamp trainees were those who expressed an interest in the Bootcamp between 2014 and 2018 but did not attend or only attended 1–2 Bootcamp meetings. Applicant success rate was defined as the percentage of Proposal Bootcamp participants (or non-Bootcamp trainees) that submitted a proposal and received at least one award. Proposal success rate (or hit rate) was defined as the percentage of submitted proposals by Proposal Bootcamp participants (or non-Bootcamp trainees) that were funded/awarded. Submission rate was defined as the percentage of Proposal Bootcamp participants (or non-Bootcamp trainees) that submitted one or more proposals. Proposals per applicant was defined as the average number of proposals submitted by Proposal Bootcamp participants (or non-Bootcamp trainees). We applied upper-tailed T-tests to compare means assuming equal variances using StatPlus.

One week after the end of the Proposal Bootcamp, participants were emailed a Qualtrics survey link to qualitatively assess self-efficacy (or confidence), which is a predictor of research career persistence and research productivity [6, 29–32]. To encourage high response rates, each year one or two survey responders randomly received a $50 gift card (e.g., to the Stanford Bookstore). Response rates were a ratio of the number of survey responders to the total number of participants. We added questions (Table 2) or items to the post-Bootcamp survey over the last six years.

**Table 2:** Post-Bootcamp survey questions. The above questions were asked to participants at the end of the Proposal Bootcamp. We indicated when specific questions were asked (“Years”). In some years, specific items were added; we indicated those items with * or ** in Figs 4 and 5.

Post-Course Survey Questions	Data Presented	Years	Response Rate (%)
Please indicate your overall satisfaction with the Grant Writing Academy’s Proposal Bootcamp course.	Fig 3A	2014–2019	72
How much did your participation in the course improve the quality of your proposal?	Fig 3B	2016–2019	74
How much did your participation in the course improve the quality of your research projects or questions?	Fig 3B	2015–2019	74
How much did the Proposal Bootcamp help you achieve these goals?	Fig 4	2016–2019; * 2017–2019	76; * 73
How has your level of confidence in the following changed from your participation in the course?	Fig 5	2014–2019; * 2015–2019; ** 2016–2019	73; * 75; ** 76
What is your level of agreement with the following statements about your weekly meetings?	Figs 6 and 7A	2015–2019	76
How much did insights during the faculty review workshop(s) help you improve the following?	Fig 7B	2015–2019	72; * 67
The faculty review workshops are an important component of the course.	Fig 8A	2014–2019	67
Please rate the following regarding your experiences at the faculty review workshops(s).	Fig 8B	2015–2019	56
Rate your degree of confidence in terms of grantsmanship domains using the scale where 0 represents no confidence and 10 indicates complete confidence in your ability to successfully perform the task indicated.	Table 5	2019	75

Participants in 2019 also completed a modified version of the abbreviated Clinical Research Assessment Inventory used by Harwood et al., 2019 [6] before and after completing the Bootcamp using Qualtrics. We used the same point scale: 0 represented no confidence and 10 indicated complete confidence [6]. As described in Harwood et al., 2019 [6], we assessed the following domains: (1) conceptualizing a study (we added the task “think independently about research”); (2) designing a study; (3) funding a study; and one additional domain we added; (4) seeking and providing feedback (tasks included: ask for specific feedback; provide feedback to peers beyond grammar and spelling; seek feedback and support from mentor(s); revise drafts based on feedback). We calculated Cronbach’s coefficient α scores, which measure the internal consistency of the instrument scale, using SPSS. We used the lavaan package in R for the confirmatory factor analysis.

The Stanford University Research Compliance Office was consulted and determined the surveys to be a quality improvement project with an except status and did not require Institutional Review Board review.

## Results

### Demographics of Bootcamp participants

Since 2014, 127 graduate students, 398 postdocs (includes 14 instructors and 5 research staff), 205 faculty, and 30 grant coaches have worked together to support proposal writing in the Grant Writing Academy’s annual autumn Proposal Bootcamps (Table 3). The Bootcamp participants had diverse backgrounds (see S4 Fig), with 44% from the clinical science departments in the Stanford School of Medicine (231/525), 36% in the biosciences (excluding bioengineering and biology) or basic science departments in the Stanford School of Medicine (191/525), almost 10% from the Stanford School of Engineering, including bioengineering (46/525), 8% from the School of Humanities and Sciences, which includes biology (44/525), and two participants from the Stanford Graduate School of Education.

**Table 3:** Participants in the annual Proposal Bootcamp. Numbers of participants are indicated for each annual Bootcamp. The total* reflects unique participants. Seventeen Bootcamp participants participated in two Bootcamps, and two Bootcamp participants participated in three Bootcamps.

	2014	2015	2016	2017	2018	2019	TOTAL*
**Writers** (Students/Postdocs)	78 (28/50)	100 (34/66)	104 (17/87)	91 (16/75)	86 (20/66)	87 (13/74)	525 (127/398)
**Faculty Reviewers**	51	70	68	58	60	66	205
**Grant Coaches**	10	11	6	6	6	6	30

### Bootcamp participants had higher proposal success rates

We assessed proposal success rates for participants that had completed the Bootcamp at least one year prior (2014–2018). The Bootcamp participants had an applicant success rate of 39% and proposal success rate of 21% for external funding sources (Table 4), submitting 629 proposals and receiving 132 awards. During this same period (2014–2018), 129 graduate students and postdocs expressed an interest in the Bootcamp (non-Bootcamp trainees) but did not attend or only attended one to two Bootcamp meetings. The non-Bootcamp trainees (Table 4) had an applicant success rate of only 21% and proposal success rate of 12% for external funding sources, submitting 76 proposals and receiving nine awards. The applicant success rate and proposal success rate for Bootcamp participants were almost two times that of non-Bootcamp trainees (p-value < 0.01 and < 0.03, respectively). Bootcamp participants and non-Bootcamp trainees that submitted proposals submitted comparable numbers of proposals per applicant (Table 4, p-value > 0.05), and requested similar dollar amounts (average amount requested = $650,000; p-value > 0.05) and numbers of months (average number of months = 36; p-value > 0.05) per proposal. The Bootcamp participants were more likely to submit a proposal than the non-Bootcamp trainees as demonstrated by higher submission rates (67% vs. 33%, p-value < 0.0001).

**Table 4:** Proposal Bootcamp participants had higher success in funded proposals. Proposal outcomes for the 2014 to 2018 Bootcamp participants compared with non-Bootcamp trainees. Bootcamp participants were more likely to ultimately submit proposals (submission rate). Bootcamp participants that submitted proposals submitted similar numbers of proposals as non-Bootcamp trainees (proposals per applicant). Bootcamp participants that submitted proposals had more funded proposals (proposal success rate) and were more likely to receive at least one award (applicant success rate). T-tests were upper-tailed comparison of means, assuming equal variances.

	Proposal Bootcamp Participants	Non-Bootcamp Trainees	P-value
**Submission Rate**	**67%**	**33%**	**<0.0001**
**Proposals per Applicant**	**2.1**	**1.8**	**>0.05**
**Applicant Success Rate**	**39%**	**21%**	**<0.01**
**Proposal Success Rate**	**21%**	**12%**	**<0.03**

### Bootcamp participants were satisfied and reported improved proposals

**Fig 3:**
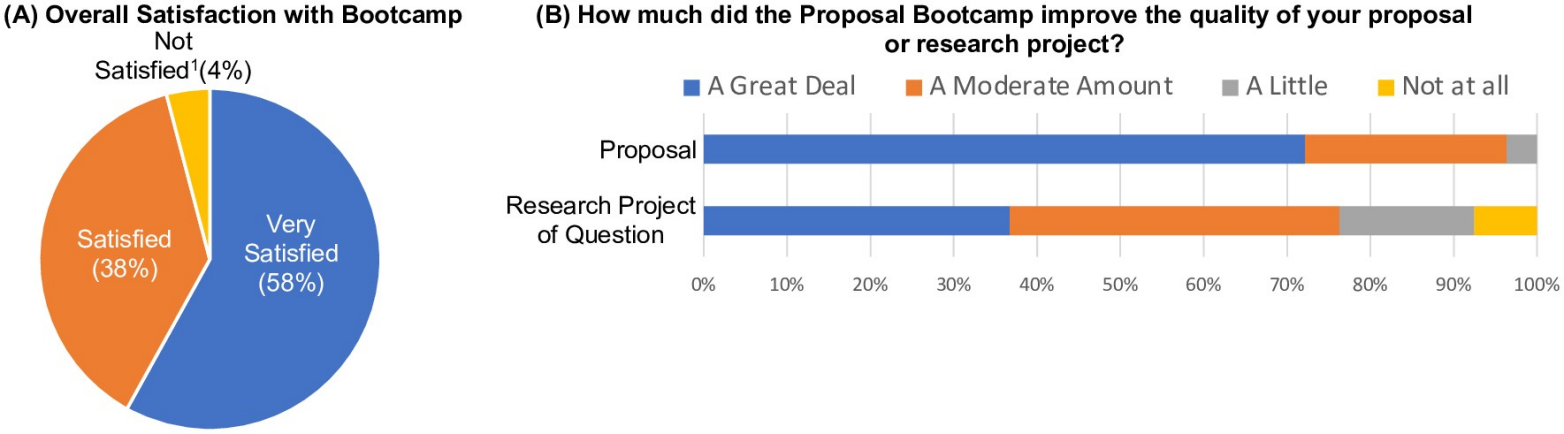
Proposal Bootcamp participants were satisfied and reported improved quality of proposals. (A) Ninety-six percent of Bootcamp participants were “Very Satisfied” or “Satisfied” with the Proposal Bootcamp. The response rate was 72%. ^1^Not Satisfied responses included “Neither Satisfied nor Dissatisfied” (3 responses), “Somewhat Dissatisfied” (4 responses), “Dissatisfied” (6 responses), and “Very Dissatisfied” (1 response). (B) Ninety-six percent of Bootcamp participants reported the Bootcamp improved the quality of their proposal “A Great Deal” or “A Moderate Amount.” Seventy-six percent of Bootcamp participants reported that the Bootcamp improved the quality of their research projects or questions “A Great Deal” or “A Moderate Amount.” The response rate was 74%.

Ninety-six percent of the Bootcamp participants were satisfied with the Bootcamp (Fig 3A). Participants reported that participation in the Bootcamp improved the quality of their proposal (96% reported “A Great Deal” or “A Moderate Amount” of improvement, Fig 3B) and their research project or questions (76% reported “A Great Deal” or “A Moderate Amount” of improvement, Fig 3B).

### Bootcamp participants gained confidence in writing proposals

In 2019, we assessed grantsmanship self-efficacy based on Harwood et al. [6], which was published in early 2019. Sixty-five Bootcamp participants completed both grantsmanship self-efficacy surveys before and after the Bootcamp. After the Proposal Bootcamp, participants were more confident in all assessed grantsmanship self-efficacy domains (Table 5): (1) conceptualizing a study (p-value < 0.0001), (2) designing a study (p-value < 0.0001), (3) funding a study (p-value < 0.0001), and (4) seeking and providing feedback (p-value < 0.0001). Cronbach’s coefficient α score revealed internal consistency for the instrument scale (α = 0.959, see S5 Fig), similar to Harwood et al. [6].

**Table 5:** Grantsmanship self-efficacy improved after the Proposal Bootcamp. The 2019 Bootcamp participants were asked to rate their degree of confidence before and after the Bootcamp in terms of 23 items across four grantsmanship domains using a 0–10 scale, 0 = no confidence, 10 = complete confidence in the ability to perform the task.

Survey Question: Rate your degree of confidence in terms of grantsmanship domains using a scale where 0 represents no confidence and 10 indicates complete confidence in your ability to successfully perform the task indicated.	Pre	Post	Change	P-value
**Conceptualize a study**	6.2	7.5	1.2	<0.0001
Articulate clear purpose				
Select suitable topic area				
Refine a problem to investigate				
Organize research ideas in writing				
Justify importance of research				
Convince reviewers that the research is worth funding				
Logical rational for research				
Relate questions to underlying theory				
Think independently about research				
**Design a study**	6.5	7.4	0.9	<0.0001
Design data analysis strategy				
Select methods of data collection				
State purpose, strengths and limits of study design				
Determine population and sample of study				
Choose appropriate research design				
Determine how each variable will be measured				
Determine adequate number of subjects				
**Fund a study**	5.2	7.0	1.8	<0.0001
Write a competitive grant				
Identify appropriate funding				
Converse with funders about the project				
Describe funding process				
**Seek and provide feedback**	6.7	7.7	1.0	<0.0001
Ask for specific feedback				
Provide feedback to peers beyond grammar and spelling				
Seek feedback and support from mentor(s)				
Revise drafts based on feedback				

**Fig 4:**
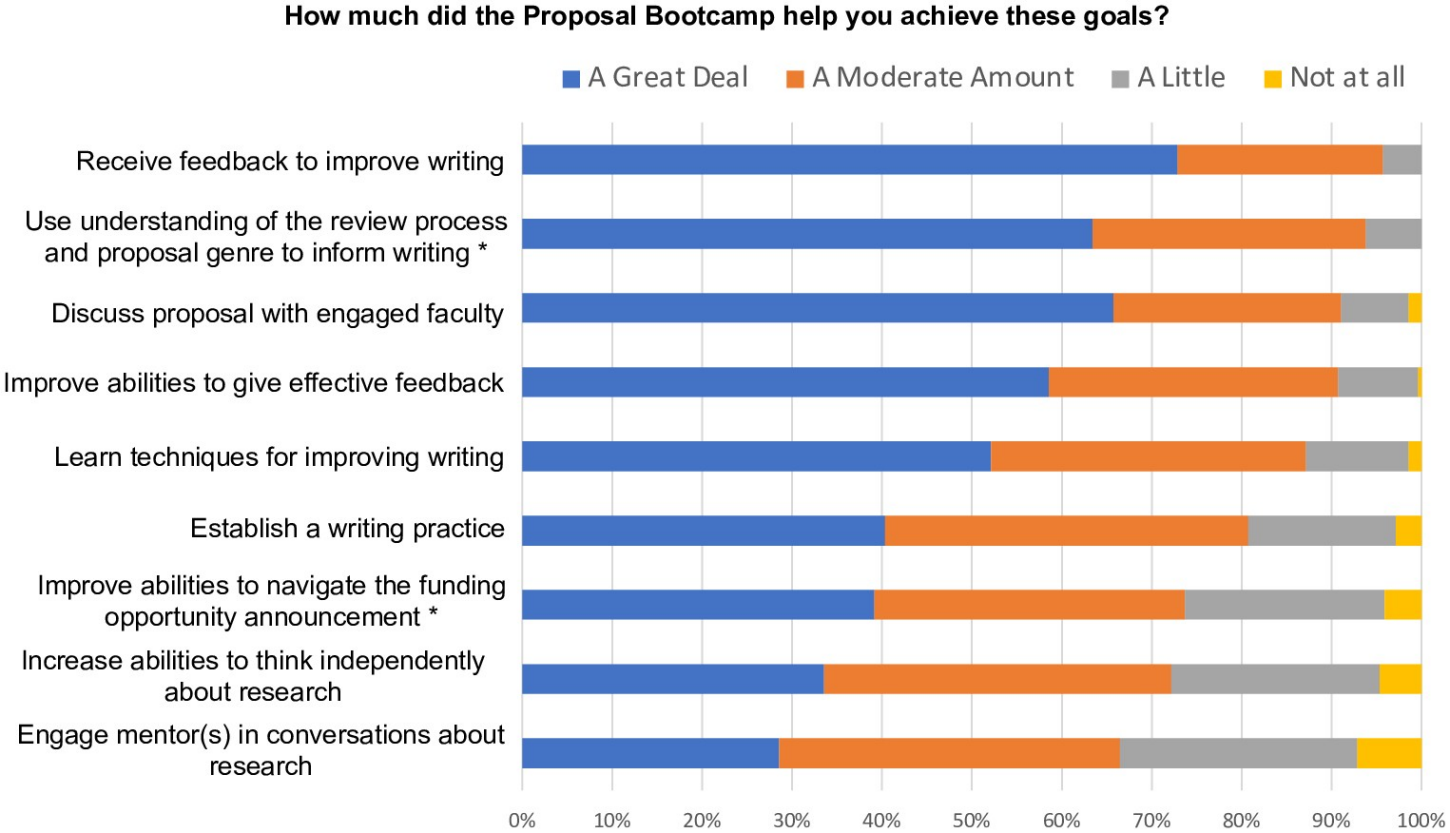
Proposal Bootcamp participants reported that they achieved learning goals. More than 93% of Bootcamp participants reported that they achieved the Bootcamp’s learning goals at least “A Little.” The response rate was 76%. *The response rate was 73%.

**Fig 5:**
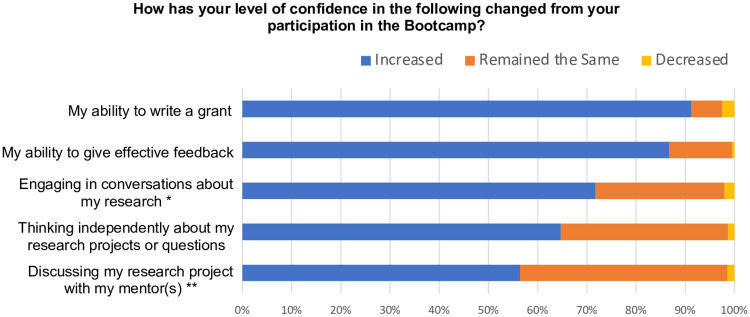
Bootcamp participants gained confidence. Ninety-one percent of Bootcamp participants reported increased confidence in grant writing abilities. The response rate was 73%. *The response rate was 75%. **The response rate was 76%.

Bootcamp participants reported learning techniques that improved their writing (87% reported “A Great Deal” or “A Moderate Amount” of improvement, Fig 4). The participants’ confidence in their ability to write a grant increased (91% reported an increase, Fig 5). Participants also reported that the Bootcamp improved their abilities to navigate funding opportunities (96% reported at least “A Little,” Fig 4) and use an understanding of the review process and proposal genre to inform their writing (100% reported at least “A Little,” Fig 4).

**Fig 6:**
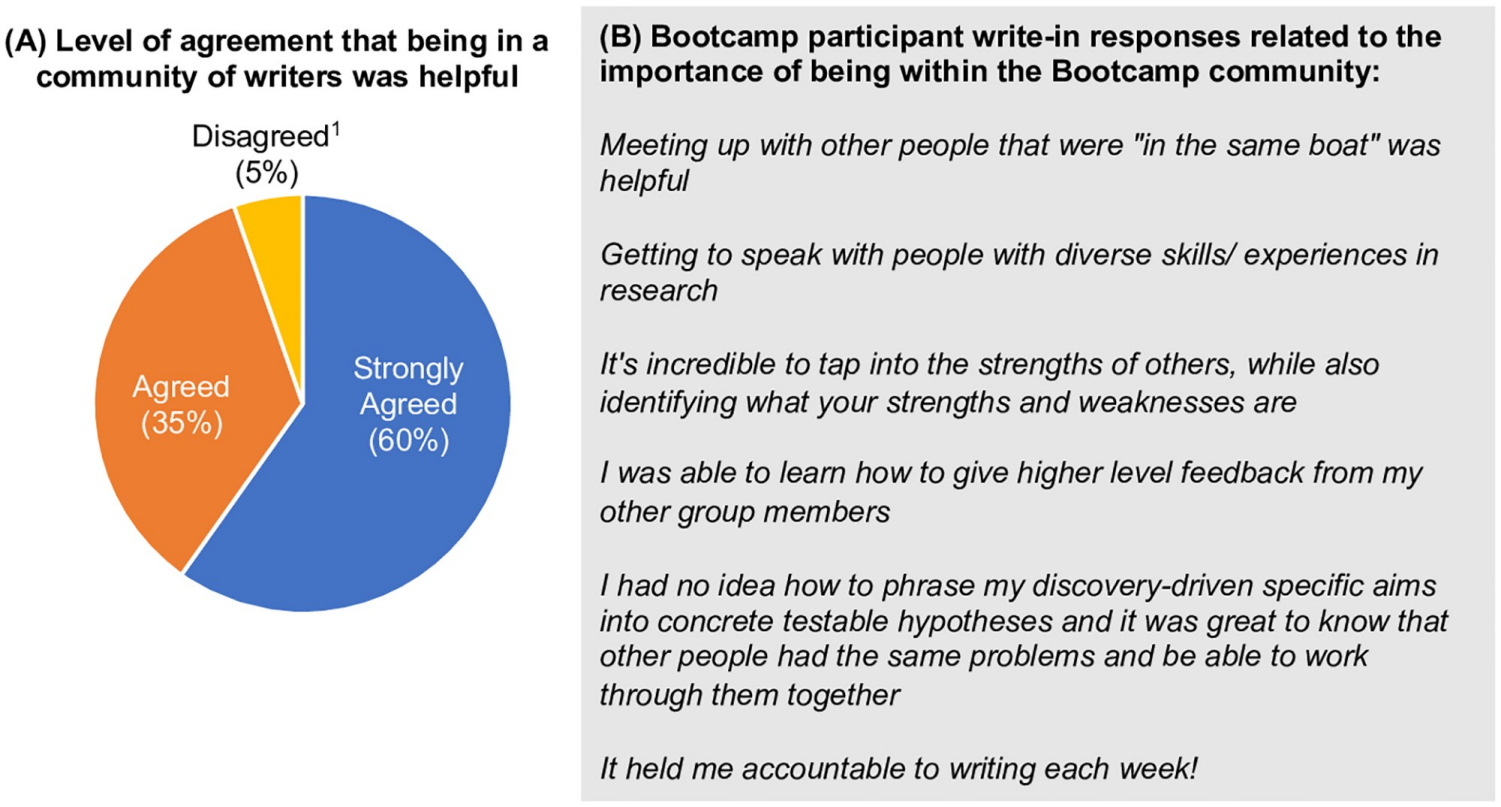
The majority of Bootcamp participants valued being in a community. (A) Ninety-five percent of Bootcamp participants reported that being in the Bootcamp community was helpful. The response rate was 76%. ^1^Disagreed responses included “Neither Agree nor Disagree” (9 responses), “Disagree” (9 responses), and “Strongly Disagree” (1 response). (B) Bootcamp participant write-in survey responses for the question “*What about this course was especially useful*?” highlighted the importance of the Bootcamp community.

Participants reported that the Bootcamp increased their abilities (72% reported “A Great Deal” or “A Moderate Amount” of increase, Fig 4) and confidence (65% reported an increase, Fig 5) in thinking independently about research projects or questions. Bootcamp participants also reported increased opportunities to engage mentor(s) in conversations about research (93% reported at least “A Little” amount of increase, Fig 4) and to discuss their research proposals with engaged faculty (91% reported “A Great Deal” or “A Moderate Amount” of increase, Fig 4). The majority of Bootcamp participants also reported increased confidence in engaging others in conversations about their research (72% reported an increase, Fig 5) and discussing their research project with their mentor(s) (56% reported an increase, Fig 5). The Bootcamp participants also valued being in a community (95% “Strongly Agreed” or “Agreed,” Fig 6).

### Bootcamp participants reported that obtaining feedback from both peers and faculty improved their proposals

**Fig 7:**
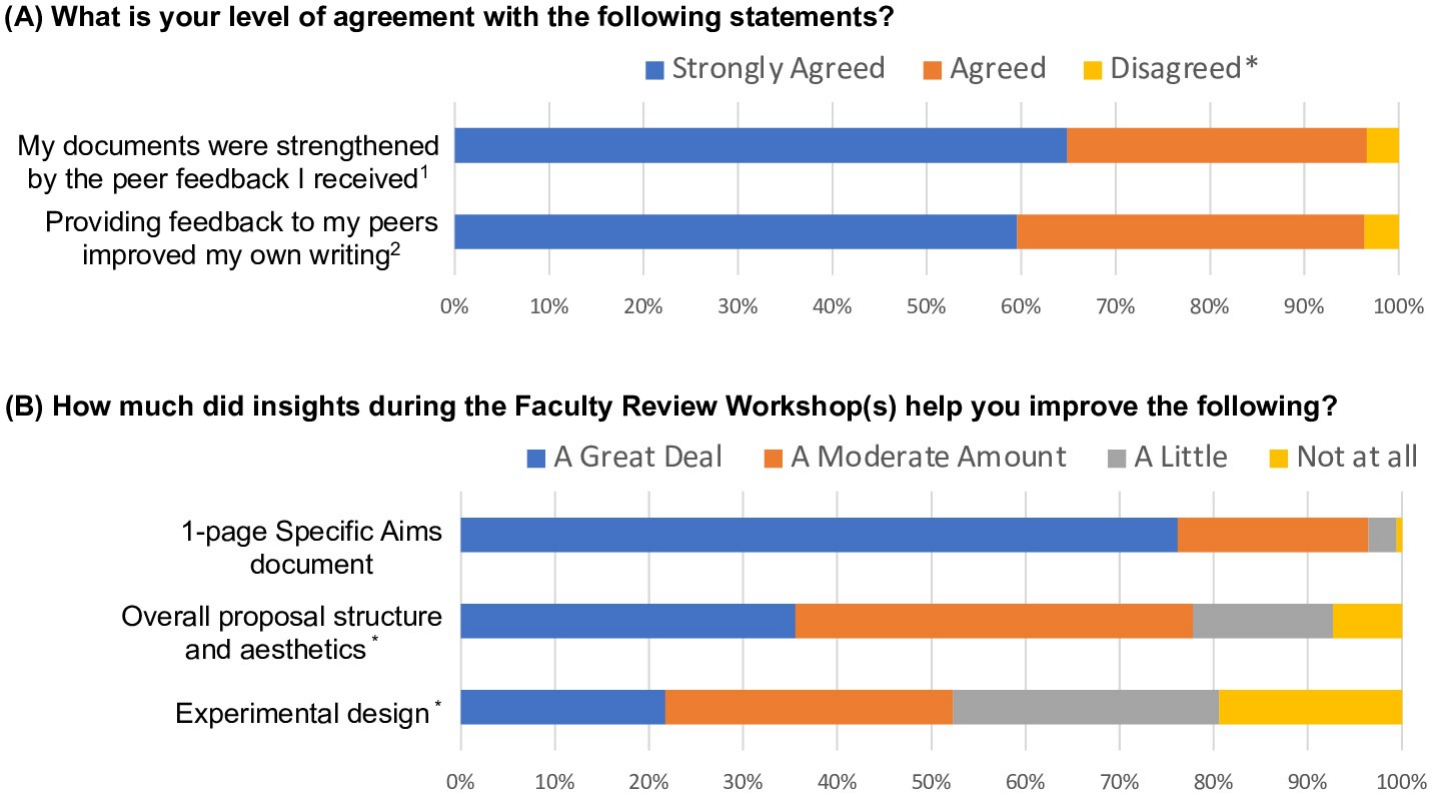
Proposal Bootcamp participants reported peer and faculty feedback improved proposals. (A) Bootcamp participants brought drafts of proposal documents to weekly meetings. Peers gave and received feedback through a structured format. Ninety-six percent of Bootcamp participants reported that this process strengthened their documents and improved their writing. The response rate was 76%. ^1^Disagree responses included “Neither Agree nor Disagree” (5 responses), “Disagree” (5 responses), and “Strongly Disagree” (2 responses). ^2^Disagree responses included “Neither Agree nor Disagree” (5 responses), “Disagree” (6 responses), and “Strongly Disagree” (2 responses). (B) As a result of the faculty review workshops, 97% of Bootcamp participants reported improved one-page Specific Aims document “A Great Deal” or “A Moderate Amount”. The response rate was 72%. *The response rate was 67%.

**Fig 8:**
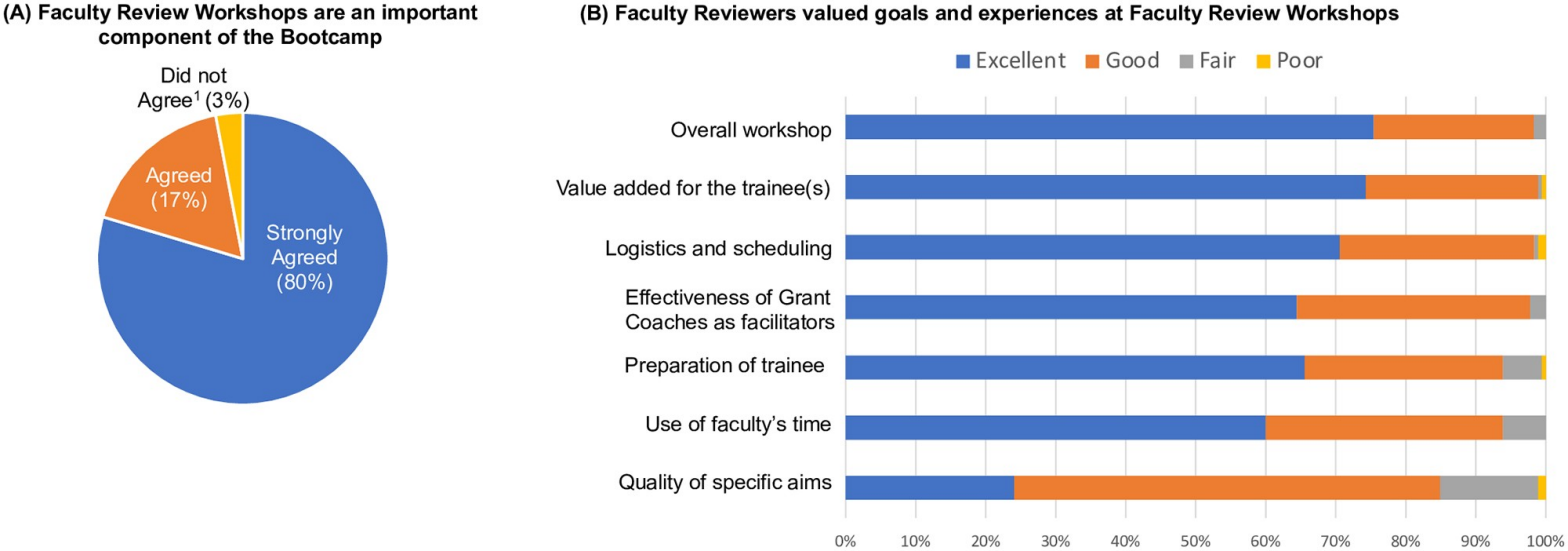
Faculty review workshops were valued by Bootcamp participants and faculty reviewers. (A) Ninety-seven percent of Bootcamp participants reported faculty review workshops were an important component of the Bootcamp. The response rate was 67%. ^1^Did Not Agree responses included “Neither Agree nor Disagree” (7 responses) and “Disagree” (4 response). (B) Ninety-eight percent of faculty reviewers rated the faculty review workshop as “Excellent” or “Good” overall. The response rate was 56%.

Bootcamp participants reported that the feedback received as part of the course improved their writing (96% reported “A Great Deal” or “A Moderate Amount” of improvement and 4% reported “A Little” improvement, Fig 4). Feedback from peers strengthened the Bootcamp participants’ proposal documents (97% “Strongly Agreed” or “Agreed,” Fig 7A). Write-in comments at the end of the Bootcamp surveys included statements such as “*the peer-review process helped me see my proposal through the eyes of a critical reviewer*.” Feedback from the faculty at the faculty review workshops improved the Bootcamp participants’ one-page Specific Aims (96% reported “A Moderate Amount” or “A Great Deal,” Fig 7B). Additionally, Bootcamp participants reported that the faculty review workshops improved the overall proposal structure and aesthetics (93%) and experimental design (81%) at least “A Little,” even though the faculty reviewers only provided feedback after reading the Bootcamp participant’s one-page Specific Aims. Write-in comments on the Bootcamp surveys included “*the faculty review workshops are fantastic and maybe a once-in-a-life kind of experience*” and “*getting varied feedback from faculty unfamiliar with my project was extremely useful and helped me to see flaws in my logic that I would have overlooked otherwise*.” Participants were satisfied with the faculty review workshops (96% reported being “Very Satisfied” or “Satisfied,” 4% reported being “Neither Satisfied nor Dissatisfied” or “Dissatisfied”) and rated the workshops as an important component of the course (97% “Strongly Agreed” or “Agreed,” Fig 8A).

### Bootcamp participants perceived that their own proposals were improved as they gave feedback to peers

Bootcamp participants reported increased abilities (91% reported “A Great Deal” or “A Moderate Amount” of increase Fig 4) and confidence in giving effective feedback (87% reported that their confidence increased; 13% reported that their confidence remained the same, Fig 5). Bootcamp participants (96%) “Strongly Agreed” or “Agreed” that providing feedback to peers improved their own writing (Fig 7A). Write-in comments on the end of the Bootcamp surveys indicated that the Bootcamp gave “*a lot of chance[s] for reviewing other people’s writing*, *during which you can realize the problem you also have*. *You have the feeling of a reviewer*. *This is super helpful*. *When you begin to have a reviewer’s point [of view]*, *you can understand your own problem*.”

### Faculty valued the Bootcamp’s goals and experience

Since 2014, 205 faculty have participated in an average of 2.5 faculty review workshops. Forty-two percent of the faculty reviewers participated in multiple years. Demographic information for the faculty reviewers is shown in S4 Fig. Overall, 98% of faculty rated the faculty review workshops as “Good” to “Excellent” and a good use of their time (94% rated as “Good” or “Excellent,” Fig 8B). The Bootcamp participants were prepared (94% rated as “Good” or “Excellent,” Fig 8B) and had high-quality specific aims (85% rated as “Good” or “Excellent,” Fig 8B). Write-in comments on surveys from faculty included the following: *“It was my pleasure to take part and I’d be glad to do it again”; “The grant writing workshop was fun”; “It was very valuable for the trainees”; “I learned a lot from working with the postdocs and graduate students”*.

## Discussion

Our goals in creating the Grant Writing Academy’s Proposal Bootcamp included: providing multi-level feedback (peer and faculty) to graduate students and postdocs to improve research proposals; increasing the Bootcamp participants’ confidence in developing and submitting research proposals; and increasing faculty engagement in the proposal development process. We found that our Proposal Bootcamp participants had higher applicant and proposal success rates, and that participants were more likely to submit an external proposal than non-Bootcamp trainees (Table 4). Bootcamp participants and non-Bootcamp trainees that submitted proposals, interestingly, submit similar numbers of proposals (Table 4) suggesting the higher success rates were not due to Bootcamp participants submitting more proposals. Bootcamp participants also gained grantsmanship self-efficacy (confidence) in grant writing abilities (Fig 5, Table 5). Additionally, 205 faculty reviewers (Table 3) provided feedback to Bootcamp participants’ in valued faculty review workshops (Fig 8).

Bootcamp participants reported that providing feedback to peers improved their writing and that their documents were strengthened by the peer feedback they received (Fig 7A). Empowering the Bootcamp participants to give feedback to their peers during the proposal writing process enabled our annual cohort to be much larger than possible if only experts, e.g., faculty or grant writers, provided feedback. Importantly, we found our grantsmanship self-efficacy (Table 5) were similar to other much smaller programs, such as the <30 person cohorts of the NIH National Research Mentoring Network grantsmanship coaching programs [6].

The Bootcamp participants (Fig 8A) and faculty (Fig 8B) valued the faculty review workshops, which required neither preparation nor follow-up on the part of the faculty members. The faculty reviewers reported Bootcamp participants were prepared (94% rated their preparation as “Good” or “Excellent,” Fig 8B). Faculty reviewers noted the majority of the one-page NIH-style Specific Aims were of good or fair quality (Fig 8B) at faculty review workshops held during the middle of the Bootcamp (week 3–4 or 5–6 of the 8-week Bootcamp). In future Bootcamps, it would be interesting to compare the faculty reviewers’ ratings of the quality of the Specific Aims with future measures of proposal outcomes, such as the proposal success rate. Similarly, we wonder whether the participants whose Specific Aims were rated as a higher quality received more feedback from their mentor/supervisor, although all mentors were instructed to provide feedback to the Bootcamp participant before the faculty review workshops. Importantly, faculty were provided only the one-page Specific Aims, but many Bootcamp participants reported that the faculty feedback improved the overall proposal structure and aesthetics as well as the proposal’s experimental design (Fig 7B). This finding suggests that our approach of having the faculty reviewers read only the one-page Specific Aims was sufficient for enabling high-quality feedback with little time investment.

Our Proposal Bootcamp participants had a 39% applicant success rate (Table 4). The Stanford Electronic Research Administration System underestimates submitted proposals because it does not include internally funded proposals, such as institutional NRSA fellowships (e.g., T32, Stanford has 69 T32 awards), K Awards (e.g., K12 or KL2) or Stanford-specific fellowships (e.g., Stanford’s Berry Fellowship, Dean’s, and Bio-X Fellowships). Additionally, proposals submitted by trainees who have left Stanford University are also missed. We estimate that our proposal outcomes would be higher if we considered all proposals submitted by our Bootcamp participants; however, measuring proposal outcomes for all submitted proposals is time consuming and imprecise because it often relies on outcomes reported by the trainee.

### Lessons learned

We are continuing to revise the Bootcamp curriculum to augment its learning goals based on feedback from Bootcamp participants, faculty reviewers, Bootcamp participants’ mentors, grant coaches, and other stakeholders. The core components of the Bootcamp, including the structured feedback format using peer-review rubrics/worksheets, weekly small group meetings led by a grant coach, and the faculty review workshops have changed minimally over the last six years. We have made the following changes based on feedback from Bootcamp participants.

In the post-Bootcamp survey, several participants in the first Proposal Bootcamp cohort stated that more time for peer review would be helpful. Thus, as a significant change to the weekly meetings, we reduced the time spent on lectures delivered by the grant coach from 30–45 minutes to less than 15 minutes (mini lectures, Table 1). We created short videos to deliver additional content online using a flipped classroom model. We also now encourage Bootcamp participants to read their peer drafts at the weekly small group meetings instead of exchanging and reading these documents outside of the weekly meeting times. We found that most peer-review groups were only reading the drafts during the weekly meeting times and the exchange of documents outside of the weekly meetings was difficult to manage. To enable draft reading and to prioritize feedback during the weekly meetings, for the 2017 cohort and beyond, we grouped peers into groups of three instead of four, which allowed more time for each peer review. After the first year, we also revised the peer-review schedule (Table 1) to include peer reviews of the research plans in the first half of the Bootcamp instead of only in the later part of the Bootcamp. We have found that the new schedule better enables Bootcamp participants to juggle working on several parts of the application instead of only thinking about the research plan near the end of the Bootcamp.

As feedback, graduate students in the 2014 cohort strongly encouraged that the Bootcamp start during the first week of Stanford’s Autumn Quarter rather than during the last weeks of summer. To compensate for the loss of time (the 2014 Bootcamp was changed from 13 weeks to 9 weeks in 2015, and is now 8 weeks), after the first year, we required the Bootcamp participants to bring a draft of their one-page Specific Aims to the first week of the Bootcamp. We typically offer a lecture on how to write Specific Aims 1–2 months before the start of the course and have a similar 30-minute online lecture available. Similarly, we also often offer optional two-hour Specific Aims workshops before the start of the Bootcamp, where participants follow the same peer-review format used in the Bootcamp to peer review drafts of their one-page Specific Aims.

Over the years, we have significantly improved the delivery of the Bootcamp assignments to participants. In the first year, we emailed the assignments, but later utilized an extensive course website (using Canvas, the course management system adopted by Stanford). Our website describes the weekly writing assignments; lists readings, for example, chapters in *The Grant Application Writer’s Workbook* [33] or *A Practical Guide to Writing a Ruth L*. *Kirschstein NRSA Grant* [34], and videos; provides links to other optional readings, videos, or resources; and includes weekly questions for the Bootcamp participant to reflect on their experiences in the Bootcamp.

### Areas for further curricular refinement

Motivating a current area of focus for improvement, roughly 15% of the Bootcamp survey responders reported dissatisfaction with the amount of feedback provided by their primary mentor/supervisor during the Bootcamp. We view the primary mentor/supervisor as a critical partner in guiding the student or postdoc in developing and refining their proposal. The primary mentor/supervisor should provide scientific feedback to ensure that the student/postdoc’s ideas are sound and on the right track. Since 2015, we have directly emailed the primary mentors/supervisors of Bootcamp participants during the first week of the Bootcamp, requesting that they read and provide timely feedback on the Bootcamp participants’ one-page Specific Aims draft before the faculty review workshops. We are now considering providing additional guidance for the Bootcamp participant on how to engage their mentors, co-mentors, or collaborators, as suggested by prior Bootcamp participants; we may also provide evidence-based mentor and mentee trainings for optimizing mentoring relationships [35]. Additionally, because the interaction between the Bootcamp participant and mentor goes both ways, we will survey primary mentors/supervisors to identify strategies to better augment this exchange.

We are continuing to address the issue of attrition with postdocs in the Bootcamp, which can affect the weekly meeting community. The Bootcamp graduate students receive 2 units of credit; thus, attrition is less of an issue for them. However, postdocs receive no credit. In 2015, we required a deposit ($75 check) when the postdoc enrolled in the Bootcamp and refunded this money once the postdoc had attended five or more weekly meetings, but this approach was logistically difficult. In later years, we provided the course textbook (*The Grant Application Writer’s Workbook*, [33]) free to any Bootcamp participant that attended seven of the eight weekly meetings. Attrition has decreased with approximately 80% of Bootcamp participants now attending seven of the eight weekly meetings (2018 and 2019 cohorts). We are continuing to explore the cause of attrition (e.g., Are the students/postdocs signing up before they are ready to write a proposal? Are they overwhelmed by the schedule? Are they changing their career goals based on the experience? Is the primary mentor encouraging the student/postdoc to write a proposal?) to better develop strategies to support the proposal writers at the appropriate time. We are also focusing on methods to better communicate that joining the Bootcamp community is advantageous but also requires each participant to be a good community member, i.e., requires full participation and attendance to the weekly meetings.

## Conclusions

The Proposal Bootcamp is a scalable model that accommodates large numbers of trainees, provides opportunities for feedback from multiple peer and faculty reviewers, and increases the participants’ confidence in developing and submitting research proposals. We increased faculty engagement in the proposal development process through faculty review workshops. We are currently collaborating with both the Stanford School of Engineering and the Stanford School of Earth, Energy, and Environmental Sciences to offer the Bootcamp to postdocs writing non-health proposals, such as NSF proposals. We believe that the Proposal Bootcamp’s core components, including using a structured feedback format with peer-review rubrics/worksheets, recruiting and training non-expert grant coaches to lead weekly small group meetings, and engaging faculty in short but impactful faculty review workshops, can be implemented at various types of institutions, including under-resourced settings, and will have a substantial positive impact in increasing the fundability of research proposals and participants’ confidence in developing proposals.

For more information, please see our website at http://grantwriting.stanford.edu.

## Supporting information

S1 Fig Research strategy rubric. (TIF)

S2 Fig Career development rubric. (TIF)

S3 Fig NIH biosketch rubric. (TIF)

S4 Fig Demographic information for Proposal Bootcamp and faculty review workshop participants. Bootcamp participants (2014–2019) were primarily from the Clinical Sciences and Basic Sciences Departments in the School of Medicine or the Biosciences Programs at Stanford. *Bootcamp participants also included those from Stanford Graduate School of Education (two participants) and Carnegie’s Department of Plant Biology (one participant). One faculty reviewer was from the Stanford School of Earth, Energy, and Environmental Sciences. Primary affiliation is indicated for participants and faculty.
(TIF)

S5 Fig Internal consistency coefficients and factor loading for grantsmanship self-efficacy variables. Proposal Bootcamp participants from 2019 (n = 65) self-reported pre and post item scores = 0–10 from "no confidence" to "complete confidence." Standardized internal consistency coefficients, or Cronbach’s α scores, range from 0 to 1.0 and indicate internal consistency between the items. Factor loadings, which measure variation between each item to each factor, are moderate. All factor loadings are significant at P<0.0001.
(TIF)
