# Supplementary figures and images for: Biosciences Proposal Bootcamp: Structured peer and faculty feedback improves trainees’ proposals and grantsmanship self-efficacy
Source: PLoS One. 2020 Dec 28;15(12):e0243973. doi: 10.1371/journal.pone.0243973 (PMC7769268; doi:10.1371/journal.pone.0243973)

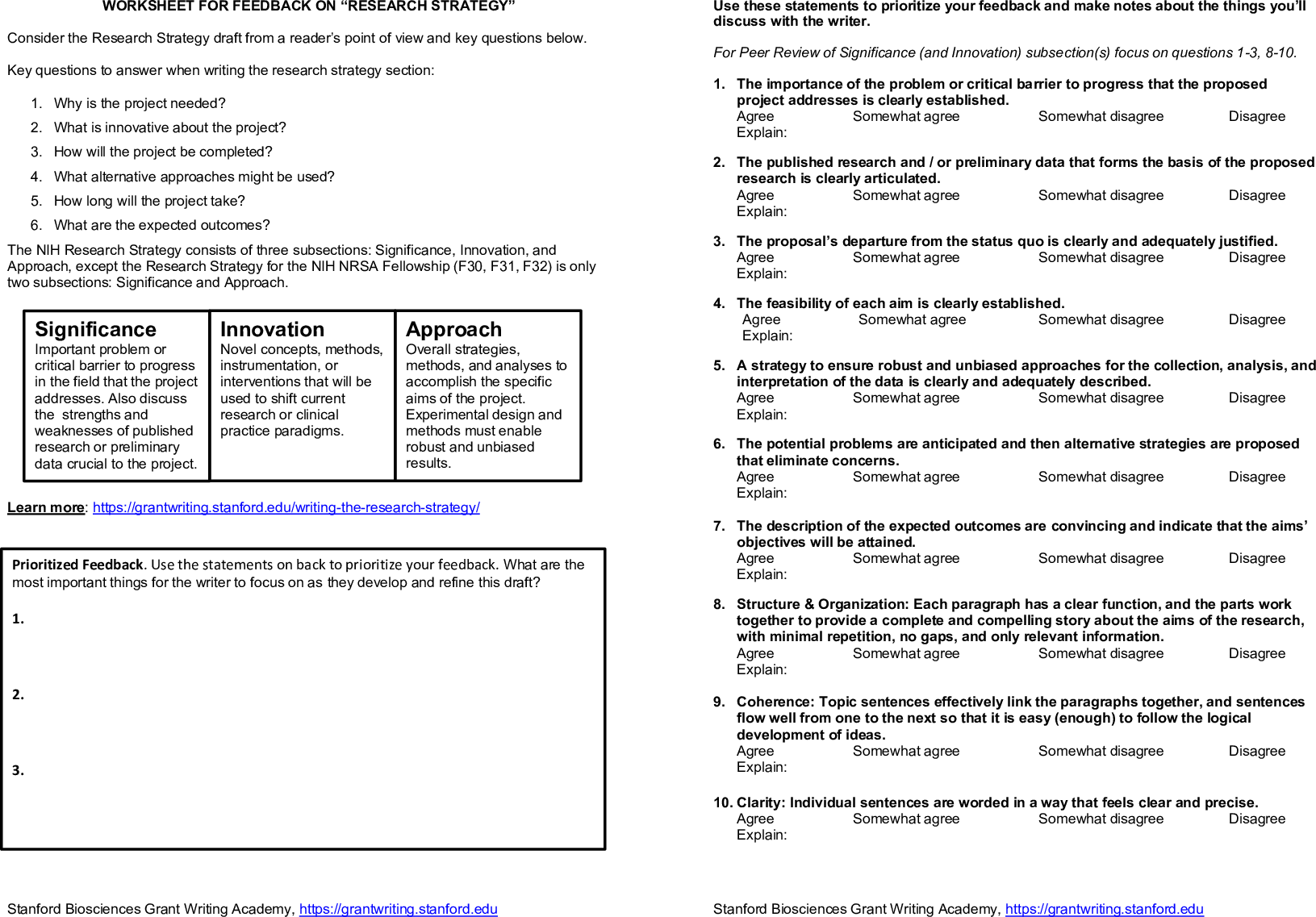

Supplement: S1 Fig — (TIF) [file pone.0243973.s001.tif]

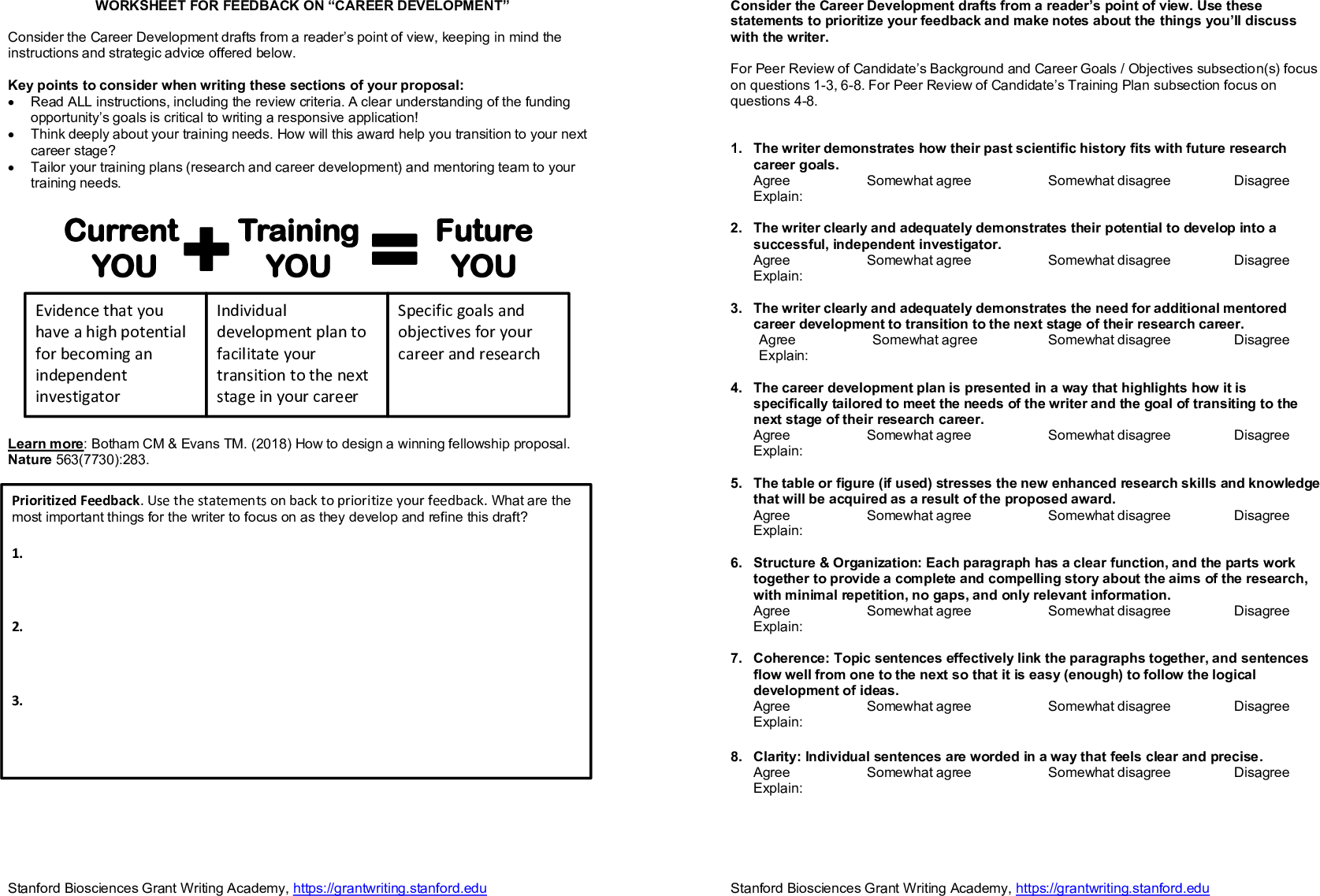

Supplement: S2 Fig — (TIF) [file pone.0243973.s002.tif]

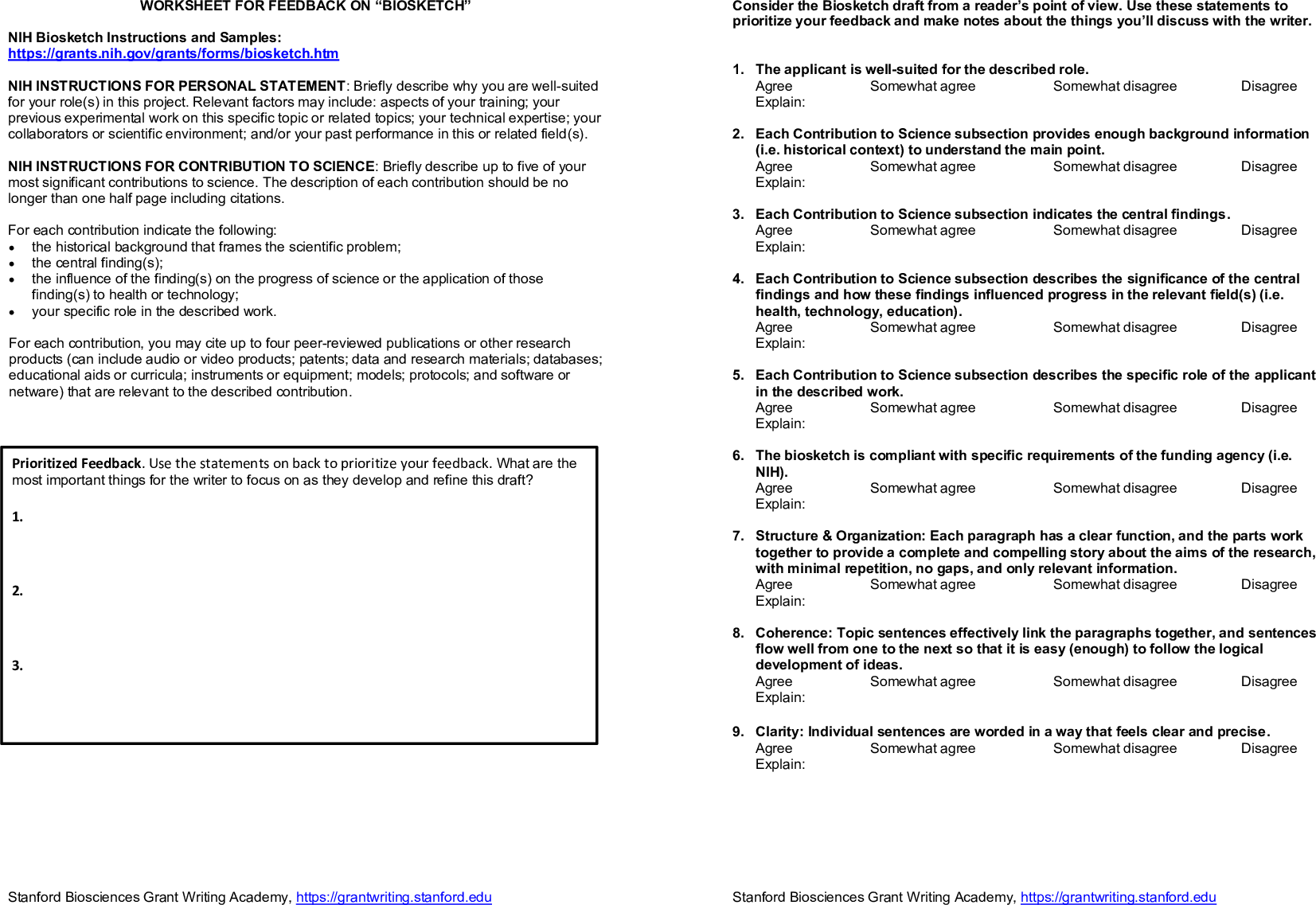

Supplement: S3 Fig — (TIF) [file pone.0243973.s003.tif]

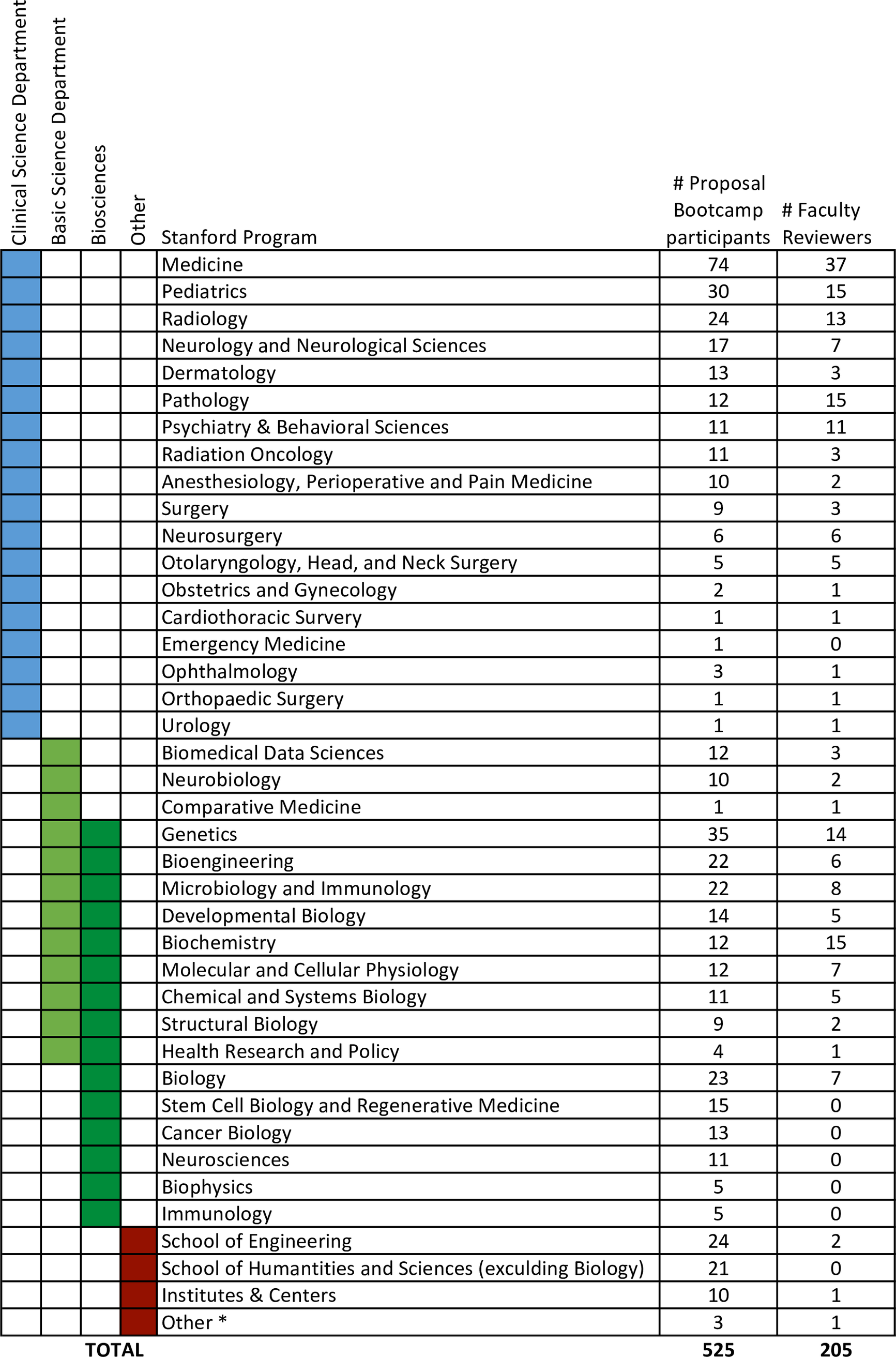

Supplement: S4 Fig — Bootcamp participants (2014–2019) were primarily from the Clinical Sciences and Basic Sciences Departments in the School of Medicine or the Biosciences Programs at Stanford. *Bootcamp participants also included those from Stanford Graduate School of Education (two participants) and Carnegie’s Department of Plant Biology (one participant). One faculty reviewer was from the Stanford School of Earth, Energy, and Environmental Sciences. Primary affiliation is indicated for participants and faculty. (TIF) [file pone.0243973.s004.tif]

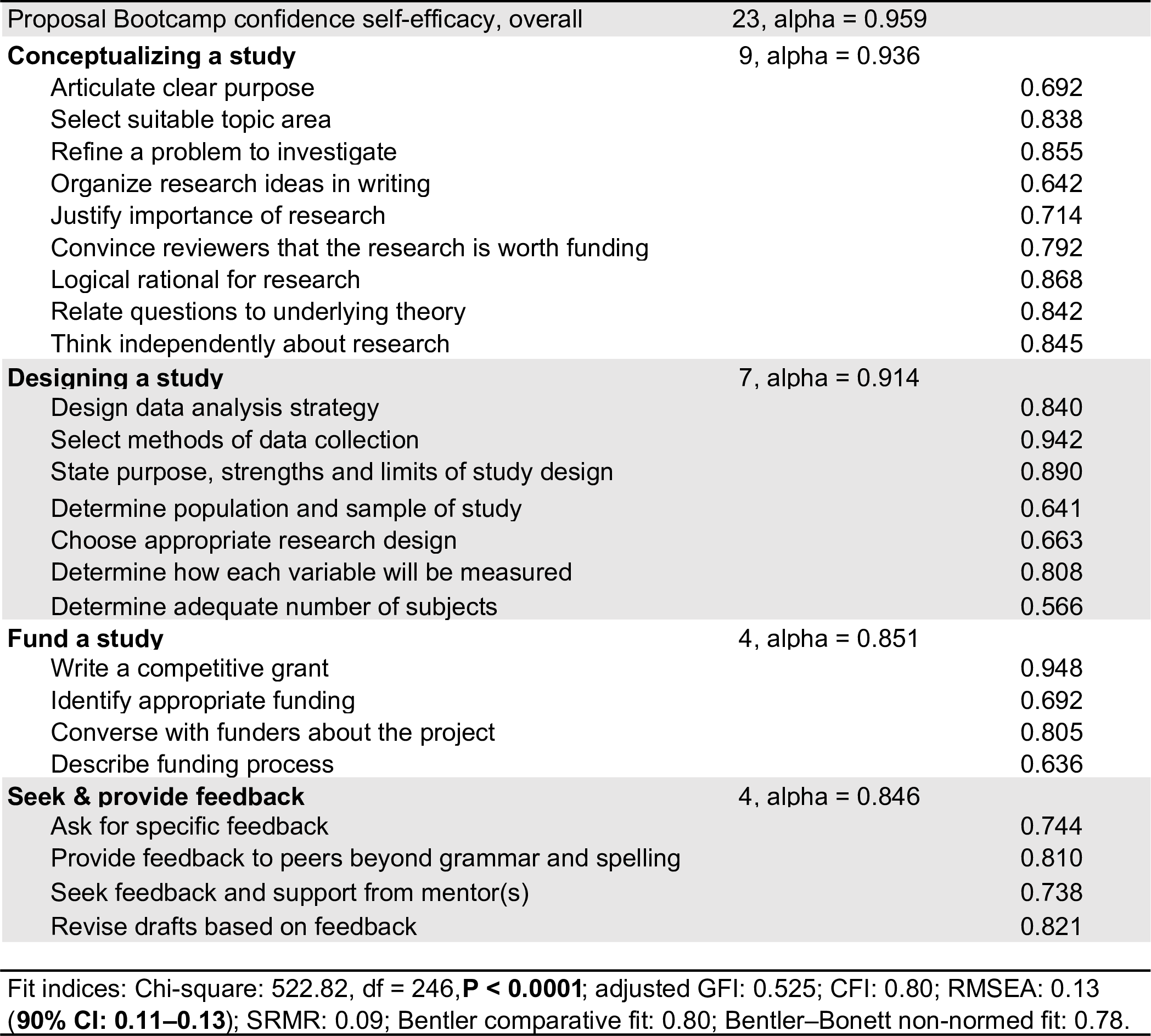

Supplement: S5 Fig — Proposal Bootcamp participants from 2019 (n = 65) self-reported pre and post item scores = 0–10 from "no confidence" to "complete confidence." Standardized internal consistency coefficients, or Cronbach’s α scores, range from 0 to 1.0 and indicate internal consistency between the items. Factor loadings, which measure variation between each item to each factor, are moderate. All factor loadings are significant at P<0.0001. (TIF) [file pone.0243973.s005.tif]
